# Seeking synergy for novel weight- and glucose-lowering pharmacotherapy and exercise training in heart failure patients with preserved ejection fraction

**DOI:** 10.1093/eurheartj/ehad856

**Published:** 2024-01-08

**Authors:** Tim Kambic, Carl J Lavie, Thijs M H Eijsvogels

**Affiliations:** Department of Medical Sciences in Sport, Faculty of Sport, University of Ljubljana, Gortanova ulica 22, Ljubljana 1000, Slovenia; John Ochsner Heart and Vascular Institute, Ochsner Clinical School, The University of Queensland School of Medicine, New Orleans, LA, USA; Department of Medical BioSciences, Exercise Physiology Research Group, Radboud University Medical Centre, Nijmegen, The Netherlands

Heart failure (HF) with preserved ejection fraction (HFpEF; EF > 50%) accounts for more than 50% of new HF diagnoses, and the prevalence is increasing due to the ageing population and the rising prevalence of obesity.^1,2^ Heart failure with preserved ejection fraction is associated with multisystem structural and functional abnormalities, contributing to a reduced physical functioning, exercise capacity, and quality of life (QoL), which subsequently increases the risk for sarcopenic obesity, frailty, unplanned hospitalizations, and mortality.^1,3^

## Benefits of exercise training

Exercise training (ET) was the only effective treatment strategy to improve health outcomes for patients with HFpEF for many decades. Improvements in maximal and submaximal aerobic capacity, muscle strength, skeletal muscle mass, fat mass, QoL, and disease symptoms were observed following completion of supervised ET programmes. These exercise-induced health benefits were also associated with a decreased risk for hospitalizations and mortality.^2–4^ Therefore, participation in an outpatient cardiac rehabilitation programme has currently a level 1A recommendation in international guidelines.^2,4^

## The new dawn of efficacious pharmacotherapy in heart failure with preserved ejection fraction

Heart failure with preserved ejection fraction treatment options remained limited for decades and predominantly targeted the reduction of symptoms (i.e. congestion) and treatment of comorbidities (i.e. hypertension and coronary artery disease).^1,2^ In recent years, the benefits of sodium–glucose cotransporter 2 inhibitors (SGLT2i; dapagliflozin and empagliflozin) for patients with HFpEF were explored based on promising findings from randomized controlled trials in patients with diabetes.^5^ A recent meta-analyses revealed that the use of SGLT2i induces a 26% risk reduction of HF hospitalization and a 20% risk reduction of cardiovascular (CV) death or first HF hospitalization in patients with HFpEF. The proportion of serious adverse effects was similar between intervention and placebo groups; thus, the use of SGLT2i was considered safe and well-tolerated.^6^ On the basis of these compelling evidence, dapagliflozin and empagliflozin were recently recommended as a class I, level A treatment for HFpEF in combination with already advised diuretics and pharmacotherapy for treatment of other CV comorbidities (angiotensin-converting enzyme inhibitors, angiotensin receptor blocker, beta-blockers, and mineralocorticoid receptor antagonists).^7^

Based on emerging evidence on the interplay between obesity and development of HFpEF,^1^ interventions enhancing weight loss may be another promising treatment strategy. Among available treatment options (diet, pharmacology), prescription of glucagon-like peptide-1 receptor agonists, such as semaglutide and liraglutide, may be considered as recent studies showed remarkable weight reductions (11.4%–15.1%) in overweight or obese patients with or without type 2 diabetes.^8^ The effects of semaglutide in overweight and obese patients with HFpEF were recently established in a randomized controlled trial.^9^ Patients receiving semaglutide had a greater weight reduction (−10.7%, *P* < .001) and larger improvements in QoL (+7.8 points in the Kansas City Cardiomyopathy Questionnaire; *P* < .001), 6-min walk test distance (+20.3 m, *P* < .001), and inflammation markers (C-reactive protein treatment ratio; −39%, *P* < .001) compared with the placebo group. Furthermore, the incidence of serious adverse events was lower in the semaglutide vs. placebo group (13.3% vs. 26.7%, *P* < .001) and was primarily driven by a lower incidence of major adverse CV events (2.7% vs. 11.3%, *P* < .001).^9^ The substantial effects of semaglutide on weight loss, QoL, physical functioning, and health outcomes will likely change future guidelines on the clinical management of HFpEF, similar to SGLT2i. Hence, it can be anticipated that SGLT2i and semaglutide will be prescribed in conjunction in the near future, to achieve maximal risk reduction.

## Potential risk of drug-induced sarcopenia

The prevalence of frailty and sarcopenic obesity is high (>80%) in patients with HFpEF, which limits their daily functioning and self-sufficiency and reduces exercise performance and QoL.^1,3^ Previous studies have demonstrated that SGLT2i and semaglutide may independently impact skeletal muscle tissue of HFpEF patients, potentially increasing the risk and magnitude of sarcopenia.^10^

Sodium–glucose cotransporter 2 inhibitors have insulin-independent euglycaemia effects and may lead to a decreased insulin-mediated utilization of glucose and amino acids in muscle and increased glucagon-evoked proteolysis, thereby promoting muscle catabolism. These mechanisms can lead to muscle weakness, fatigue, and muscle wasting. Such rare drug-induced symptoms were of transient nature, though, and resolved following 2 weeks after the discontinuation of SGLT2i.^10^

Semaglutide promotes glucose uptake in peripheral tissues and regulates hunger hormones towards greater satiety.^8^ Although the semaglutide-induced rapid weight loss primarily targets fat mass reduction, lean and skeletal muscle mass will also decline,^1,3^ which may increase the risk of sarcopenia and/or frailty in obese HFpEF patients. Future trials in patients with HFpEF are needed to (i) establish the proportion of lean body mass loss following semaglutide therapy, (ii) identify predictors of disproportional losses, and (iii) develop mitigation strategies for maximal preservation of lean tissue during weight loss therapy.

## Seeking synergy

**Figure 1:**
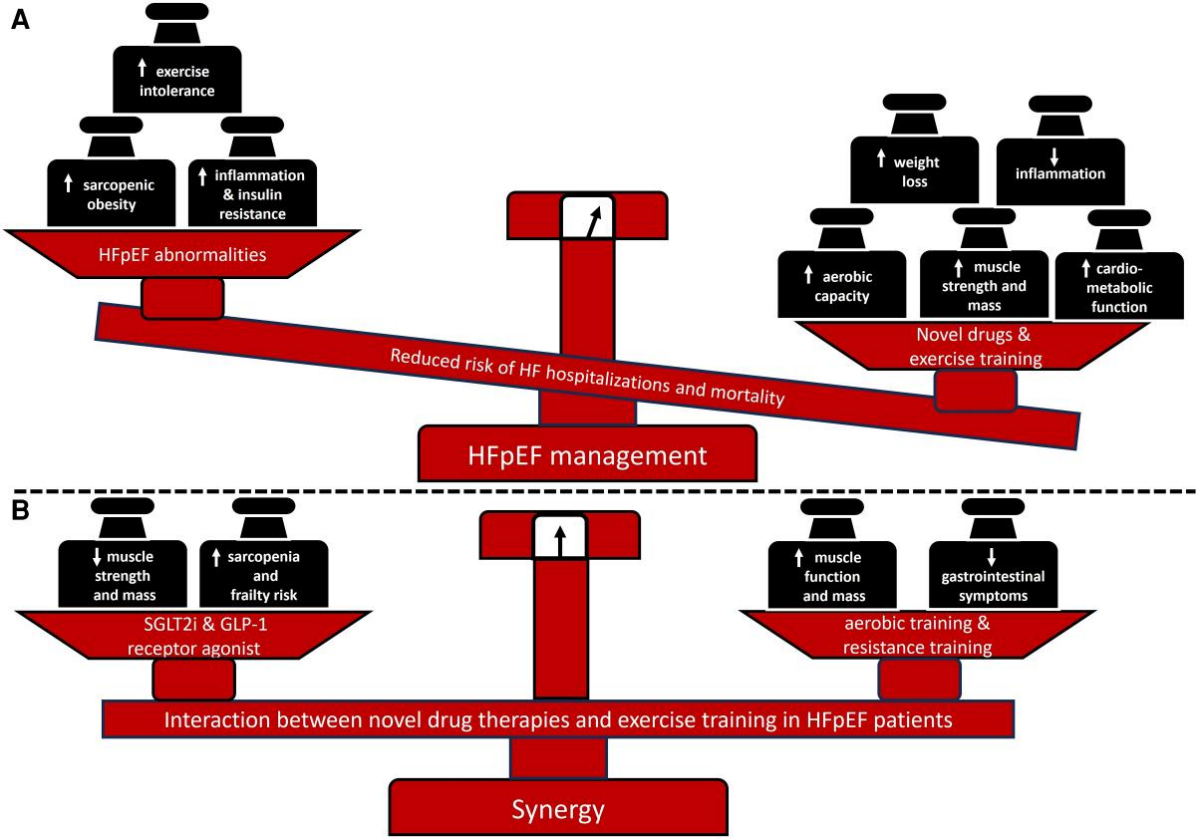
Management of heart failure with preserved ejection fraction. (*A*) Benefits of novel weight- and glucose-lowering pharmacotherapies combined with exercise training. (*B*) Risk management of potential drug-induced sarcopenia with exercise training. HFpEF, heart failure with preserved ejection fraction; GLP-1, glucagon-like peptide-1 receptor agonist; SGLT2 inhibitor, sodium–glucose cotransporter 2 inhibitors

Sodium–glucose cotransporter 2 inhibitors and semaglutide are highly effective drugs for the management of patients with HFpEF^10^ but may have deleterious effects on skeletal muscle tissue in some patients. Concurrent ET and drug therapy may mitigate the risk of sarcopenia and frailty.^2,4^ Multimodal ET should prioritize the use of moderate to high load resistance training (50%–80% of one repetition maximum) to enhance muscle mass, strength, physical functioning, and QoL (*Figure 1*).^1,2,4^ Additional aerobic training can also attenuate gastrointestinal symptoms that are often induced by semaglutide,^9^ beyond the typical improvements of physical performance. The addition of caloric restriction may further improve physical functioning^1^ and could also be a substitute for semaglutide treatment in the case of common gastrointestinal symptoms^9^ or even rapid muscle loss in HFpEF patients with sarcopenic obesity.^10^

In conclusion, the emerging clinical research of novel glucose-lowering and weight-lowering agents has provided clinicians with efficacious pharmacological options, which should be prescribed together with ET and other lifestyle interventions for maximal synergy of treatment effects for patients with HFpEF.
